# The prevalence of nine human herpesviruses in urogenital samples from young women in Finland

**DOI:** 10.1186/s12879-025-11392-6

**Published:** 2025-08-18

**Authors:** Hanna Välimaa, Tiina Eriksson, Matti Lehtinen, Ville N. Pimenoff, Mirja Puolakkainen

**Affiliations:** 1 Department of Virology, University of Helsinki, PO Box 21, 00014 Helsinki, Finland; 2 Department of Oral and Maxillofacial Diseases, Helsinki University Hospital, Helsinki, Finland; 3 Department of Infectious Diseases, Meilahti Vaccine Research Center MeVac, University of Helsinki and Helsinki University Hospital, Helsinki, Finland; 4 Faculty of Medicine, University of Tampere, Tampere, Finland; 5 Department of CLINTECH, Karolinska Institute, Stockholm, Sweden; 6 Unit of Population Health, University of Oulu, Oulu, Finland

**Keywords:** Herpes simplex virus 1, Herpes simplex virus 2, Cytomegalovirus, Epstein–Barr virus, Human herpesvirus 6, Human herpesvirus 7, Sexually transmitted infection, *Chlamydia trachomatis*, Human papillomavirus, Coinfection

## Abstract

**Background:**

Beyond herpes simplex virus (HSV), the pathogenic role and occurrence of other human herpesviruses (HHVs) in the genitourinary tract remain largely unclear. This study aimed to determine the prevalence and level of shedding of nine typical human-infecting herpesviruses in genitourinary specimens of young women.

**Methods:**

We investigated the prevalence and quantity of HHVs using qPCR in urogenital samples from 380 women participating in a community-randomized human papillomavirus (HPV) vaccination trial and in a randomized trial on the effectiveness of *Chlamydia trachomatis* screening.

**Results:**

The prevalence of Epstein–Barr virus (EBV) DNA was the most frequent finding (8.4% positive). Other herpesvirus DNA was detected less frequently: HHV-6B in 5.8%, cytomegalovirus (CMV) in 3.7%, HHV-7 in 1.6%, HSV-2 in 0.8%, and HSV-1 in 0.5% of the specimens. The varicella–zoster virus (VZV), HHV-6A, and Kaposi’s sarcoma–associated herpesvirus (KSHV/HHV-8) DNA were not detected in any of the samples. Additionally, the prevalence of EBV DNA was significantly higher among women who tested positive for *C. trachomatis* based on a nucleic acid amplification test (NAAT) compared to those who tested negative (11.3% vs. 5.7%, *p* = 0.049). Similarly, EBV DNA was detected more frequently in women with DNA from sexually transmitted microbes in their samples (*C. trachomatis*, *Mycoplasma genitalium*, HPV, or HSV), with a prevalence of 11.4% compared to 4.0% (*p* = 0.0132).

**Conclusions:**

The increased prevalence of EBV DNA among young women with a sexually transmissible pathogen coinfection suggests more frequent shedding of EBV DNA among those with a sexually transmitted infection and likely higher sexual risk-taking behavior.

## Background

Human herpesviruses (HHVs) are widespread pathogens associated with various types of diseases, ranging from relatively mild mucocutaneous infections to febrile syndromes, congenital and neonatal infections, life-threatening central nervous system (CNS) infections, and transplant complications.

Following primary infection, herpesviruses establish a lifelong latency in specific cell types, such as nerve cells and immune cells. Periodic reactivation from latency leads to either symptomatic infection or asymptomatic shedding of the virus to the mucosal surfaces. In the genital area, both herpes simplex virus (HSV) and Kaposi’s sarcoma–associated herpesvirus (KSHV/HHV-8) are transmitted through direct contact with infectious lesions or body fluids. These viruses share the same transmission route as pathogens that commonly cause sexually transmitted infections (STIs) such as human papillomavirus (HPV), *Chlamydia trachomatis*, and *Mycoplasma genitalium*.

Herpes simplex virus types 1 (HSV-1) and 2 (HSV-2) can cause vesicular and ulcerative lesions on the skin and mucosal surfaces, as well as herpetic keratitis, CNS infections, and neonatal herpes. Both types are also important etiological agents of ulcerative genital infections [1]. Alongside congenital infections and febrile lymphadenopathy, cytomegalovirus (CMV) reportedly causes anogenital ulcers, particularly in immunocompromised individuals [2]. Epstein–Barr virus (EBV), the etiologic agent of infectious mononucleosis, has also been associated with painful acute vulvar ulcerations, commonly referred to as Lipschütz ulcers, which typically occur in young sexually inactive females [3]. Kaposi’s sarcoma, linked to Kaposi’s sarcoma–associated herpesvirus (KSHV/HHV-8), can involve internal organs, the skin, and mucosal surfaces, including urogenital sites [4].

Other herpesviruses have occasionally been detected in the genital tract [5, 6]; however, their prevalence and potential pathogenicity in the genitalia remain largely undefined. The mucocutaneous manifestations of these herpesviruses include varicella and herpes zoster caused by the varicella–zoster virus (VZV), as well as roseola infantum associated with HHV-6B and HHV-7. By contrast, HHV-6A, a known neurotropic pathogen, has not been clearly linked to any mucocutaneous manifestations. Little is currently known about potential urogenital coinfections involving herpesviruses and STI pathogens.

This study aimed to investigate the prevalence and level of shedding of nine human herpesviruses in the genitourinary swabs of young women.

## Materials and methods

### Samples

The samples analyzed here represent a subset (*n* = 380) of anonymous urogenital samples collected from women participating in a community-randomized HPV vaccination trial in Finland between 2010 and 2017 [7] who simultaneously participated in a trial on the effectiveness of *C. trachomatis* screening [8] at the age of 18 or 22 in Finland in 2010–2017. The self-collected cervico-vaginal swabs rinsed in first-void urine were sent to Fimlab (Tampere, Finland) for *C. trachomatis* and *N. gonorrhoeae* testing using the Abbott RealTime CT/NG assay (Abbott Molecular, Des Plaines, IL, USA). From these samples, 186 contained *C. trachomatis* DNA. All women with a *C. trachomatis*-positive sample and their partners received free-of-cost treatment (1-g single-dose azithromycin). No gonococcal DNA was detected. Earlier, the samples had been analyzed for HPV DNA in the Department of Clinical Microbiology, Skåne University Hospital, Malmö, Sweden using methods described previously [9, 10]. HPV DNA was found in 166 samples.

**Fig. 1:**
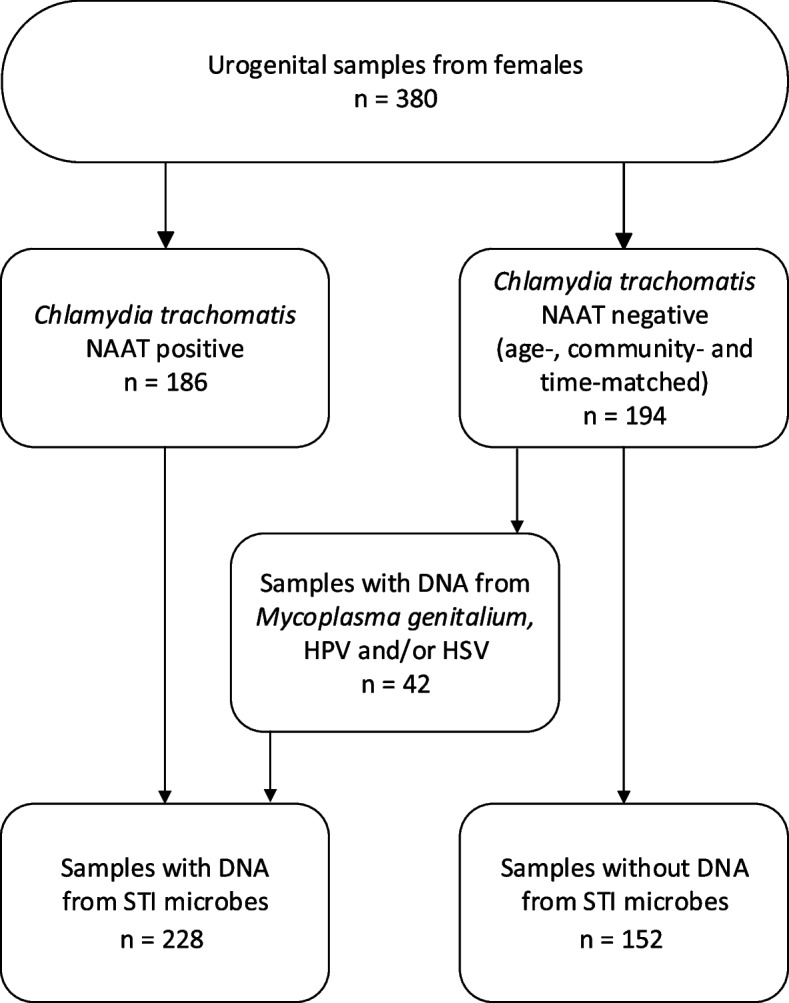
Description of the study material. HPV, human papillomavirus; HSV, herpes simplex virus; NAAT, nucleic acid amplification test; STI, sexually transmitted infection

The study material comprised urogenital samples from a total of 380 women aged 18 or 22 years old, including 186 individuals who tested positive for *C. trachomatis* using a nucleic acid amplification test (NAAT), and 194 age-, community- and time-matched *C. trachomatis* NAAT-negative controls. Among the 194 *C. trachomatis* NAAT-negative samples, 40 contained HPV (earlier data [7, 8]). In addition, in this study, two samples contained *Mycoplasma genitalium* and/or HSV DNA. These 42 samples were analyzed as the samples containing DNA from the STI microbes (Fig. 1).

### DNA extraction and real-time PCR analyses

DNA was extracted from the urogenital samples using the Maxwell®16 Viral Total Nucleic Acid Purification Kit (Promega; Madison, WI, USA). HHVs were detected through a pan-herpes quantitative multiplex real-time PCR (HER-Q) as previously described [6]. *M. genitalium* (*mgbB* gene) [11] was detected using real-time PCR [12]. To evaluate the sample quality, the presence of PCR inhibitors, the DNA integrity, and the performance of the nucleic acid extraction, the human beta-globin gene was successfully amplified in all samples analyzed [13]. A no-template control was included in each run, and it remained negative for all PCRs.

### Statistical analysis

We calculated the *p*-values for the Fisher’s exact test using IBM’s SPSS Statistics version 24 (IBM, Armonk, NY, USA), and considered *p* < 0.05 as statistically significant.

## Results

**Fig. 2:**
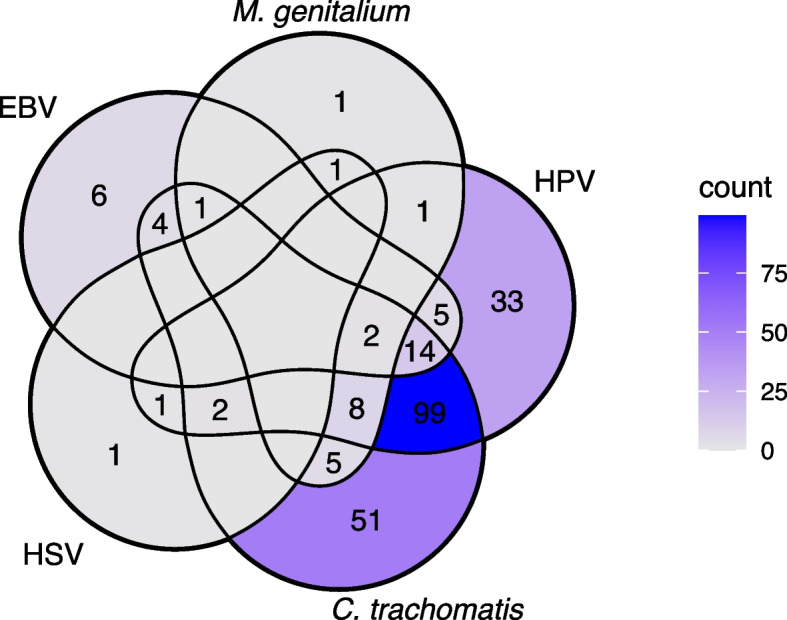
A Venn diagram illustrating the co-occurrence of EBV with any sexually transmitted pathogens (*C. trachomatis*, *M. genitalium*, HPV, and HSV). HSV represents the occurrence of either HSV-1 or HSV-2. EBV, Epstein-Barr virus; HPV, human papillomavirus; HSV, herpes simplex virus

Among the 380 specimens, any HHV DNA was detected in 17.1% (*n* = 65) of the samples. The highest prevalence of HHV was 8.4% for EBV (*n* = 32) and 5.8% for HHV-6B (*n* = 22). The prevalence of other herpesviruses in descending order were as follows: 3.7% for CMV (*n* = 14), 1.6% for HHV-7 (*n* = 6), 0.8% for HSV-2 (*n* = 3), and 0.5% for HSV-1 (*n* = 2). Neither VZV, HHV-6A nor KSHV DNA was detected. DNA from two herpesviruses was identified in nine samples: EBV/CMV, EBV/HHV-6B, and CMV/HHV-6B were each detected in two samples, while EBV/HHV-7, CMV/HHV-7, and HHV-6B/HHV-7 were each detected in one sample. Among the 48.9% (*n* = 186) of women with a positive *C. trachomatis* NAAT and the 43.7% (*n* = 166) of women with HPV DNA, an EBV coinfection was the most common finding (11.3% and 12.7%, respectively). Those 60.0% (*n* = 228) of women with at least one STI pathogen had a significantly (*p* = 0.0132) higher prevalence (11.4%) of EBV coinfections compared with the prevalence among women without any STIs (4.0%; Table 1). The co-occurrence of EBV and various STI agents is visualized in the Venn diagram in Fig. 2.

**Table 1:** Co-occurrence of human herpesviruses with sexually transmitted pathogens

	Any STI agent DNA^1^								Fisher’s exact^2^	*p* value
	Positive (*n* = 228)				Negative (*n* = 152)					
**Virus**	**n**	**%**	**95% CI**	**Median number of copies/ml**	**n**	**%**	**95% CI**	**Median number of copies/ml**		
EBV	26	11.4	7.27–15.53	2.7 × 10^3^	6	4.0	0.85–7.04	1.0 × 10^4^	0.0132	< 0.05
CMV	12	5.3	2.36–8.16	1.3 × 10^4^	2	1.3	0–3.1	1.6 × 10^3^	0.053	NS
HHV-6B	11	4.8	2.04–7.60	2.2 × 10^3^	11	7.2	3.1–11.4	2.1 × 10^3^	0.3726	NS
HHV-7	2	0.9	0–2.09	1.9 × 10^3^	4	2.6	0.1–5.2	1.6 × 10^3^	0.2228	NS
Any of the above	44	19.3	14.18–24.42	NA	21	13.8	8.3–19.3	NA	0.2105	NS
CMV and/or EBV	36	15.8	11.06–20.52	NA	8	5.3	1.7–8.8	NA	0.0017	< 0.01

The prevalence (n, %, and 95% CI) and median copy numbers/ml of specimen of human herpesviruses among women having with at least one established sexually transmitted pathogen^1^ (*n* = 228) and among those with no evidence of sexually transmitted pathogens (*n* = 152)

*CI* confidence interval, *CMV* cytomegalovirus, *EBV* Epstein–Barr virus, *HHV-6B* human herpesvirus 6B, *HHV-7* human herpesvirus 7, *HPV* human papillomavirus, *HSV* herpes simplex virus, *STI* sexually transmitted infection

^1^Included *C. trachomatis*, *M. genitalium*, HPV, HSV-1, and HSV-2 (no gonococcal DNA was detected in any specimen)

^2^Fisher’s exact test was used to calculate the statistical significance of prevalence between groups

**Fig. 3:**
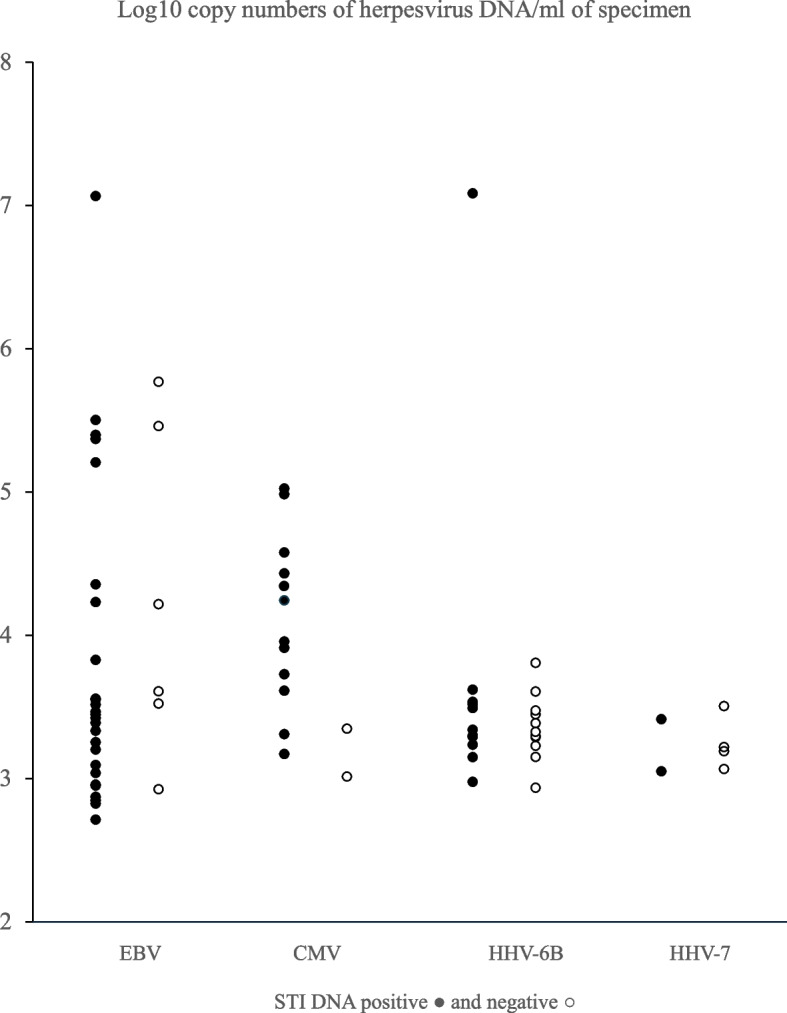
The quantity of herpesvirus DNA in urogenital samples. Samples from individuals with and without DNA from sexually transmitted pathogens (*Chlamydia trachomatis*, HPV, *Mycoplasma genitalium*, HSV-1, or HSV-2) are indicated with full circles • and open circles ○, respectively. CMV, cytomegalovirus; EBV, Epstein–Barr virus; HHV-6B, human herpesvirus 6B; HHV-7, human herpesvirus 7; HPV, human papillomavirus; HSV, herpes simplex virus; STI, sexually transmitted infection

The quantity and median copy numbers of herpesviruses in the genitourinary specimens appear in Table 1 and Fig. 3. CMV copy numbers trended higher among those positive for STI pathogens. The viral copy numbers did not differ significantly between STI pathogen DNA-positive and STI pathogen DNA-negative specimens, although the subgroups with viral DNA were rather small.

## Discussion

We observed herpesvirus DNA in 17.1% of the urogenital samples of young females simultaneously participating in an HPV vaccination trial and in a community-randomized trial on the effectiveness of *C. trachomatis* screening [7, 8]. EBV, HHV-6B, and CMV DNA were the most frequent findings, while HHV-6A, VZV, and KSHV DNA were not detected. HSV and HHV-7 DNA remained rare, while EBV DNA positivity significantly associated with the detection of a concomitant STI pathogen.

Herpesviruses are ubiquitous pathogens that establish lifelong latency with the potential for periodic reactivation. In a large cohort of Finnish pregnant women, the seroprevalence of HSV-1, HSV-2, VZV, EBV, and CMV was 45%, 11%, 98%, 95%, and 71.5%, respectively [14]. In our study, EBV DNA was detected approximately twice as often as CMV DNA (8.42% for EBV vs. 3.68% for CMV). Previously, a Swedish study found CMV (11.5%) and EBV (10.5%) in cervical cytobrush samples from healthy young Swedish women, whereas HSV-1 was detected in only 1.5%, HSV-2 in 1.4%, and VZV in none of the samples [5]. Our proportionally lower CMV DNA detection rate compared with EBV may reflect the lower CMV seroprevalence [14].

Following primary infection, HSV is intermittently shed to the genital mucosa with HSV-2 shedding occurring more frequently than HSV-1 [15, 16]. In this study, HSV-1 and HSV-2 were detected at comparable rates (0.53% and 0.79%, respectively), despite expectations of a higher HSV-2 prevalence based on existing Finnish epidemiological data [17, 18], and of a more frequent genital reactivation rate of HSV-2. Our findings suggest that HSV-1 may play a more prominent role in genital herpes among young females than previously thought.

Despite an adult seroprevalence of > 90% for HHV-6 [19] and HHV-7 [20], HHV-6B was detected in 5.8%, HHV-7 findings remained rare, and HHV-6A remained undetected, indicating infrequent genital shedding. This partly agrees with earlier findings, whereby HHV-6B DNA was detected in 6.8% and HHV-7 DNA in 79.3% of cervical samples [21]. In contrast to our findings, HHV-6A was previously observed to account for 29% of vaginal swab HHV-6 DNA findings [22]. Pregnancy can influence the reactivation and shedding of HHV-6 and HHV-7 [22–24], but it was unknown if any pregnancies occurred among our study participants. In line with earlier studies [5], VZV DNA remained undetected, supporting infrequent genital shedding despite > 95% seroprevalence [14]. Moreover, KSHV DNA was also not detected, as expected due to the low seroprevalence (< 10%) [25].

DNA from two herpesviruses was detected in nine samples (2.4% of all samples, 12.9% in samples positive for any herpesvirus). The co-prevalence of herpesviruses in genital samples was previously observed both as shedding and in symptomatic disease [5, 6, 23]. Coinfections have been detected in mucocutaneous and disseminated infections, particularly in immunocompromised individuals [26–31].

Less is known about the co-occurrence and interaction of herpesviruses with STI pathogens. STIs may promote herpesvirus reactivation and herpesviruses may influence the pathogenesis of other STI agents. Indeed, in vitro studies have demonstrated that *C. trachomatis* and HHV-6 as well as HSV-2 interact during infection and these herpesviruses can promote persistence of *C. trachomatis* [32, 33]. In our earlier study, HHV-6 DNA was slightly more common among those who provided a *C. trachomatis*–positive test of cure sample (19% vs. 10%, not significant) taken one month after the treatment [12]. In this study, however, HHV-6B DNA was equally present in STI agent–positive and negative individuals. Thus, its potential effect on promoting development of chlamydial persistence or on treatment failure remained open.

EBV and CMV transcripts have been co-detected with HPV from cervical samples, and EBV found to associate with cervical high-grade squamous intraepithelial lesions [34], but assessing the role of EBV in HPV-related carcinogenesis lay beyond the scope of this cross-sectional study. While EBV is primarily transmitted via saliva, it remains unclear how EBV reaches the urogenital tract. The detection of EBV DNA may reflect either reactivation of latent infection or the migration of EBV-bearing lymphocytes to the site of inflammation. The detection of herpesvirus DNA in the genital mucosa suggests the potential for sexual transmission, although only HSV and KSHV are well-established as sexually transmitted viruses. Sexual transmission has also been proposed for EBV and CMV [5, 35, 36], along with intrapartum transmission. These findings highlight the value of multiplex testing or NGS techniques and call for studies on the pathogenetic role of coinfections.

There are a few limitations to this study. Due to the nature of the study, neither follow-up samples nor serum samples were available for further estimating the clinical significance of herpesvirus DNA positivity. In addition, the observed herpesvirus DNA does not directly indicate that the virus is infectious nor that the infection is in an active state. Furthermore, the effect of self-sampling on DNA quantity cannot be excluded. Finally, no information on medical history, possible genitourinary symptoms, or clinical findings were available.

Our findings support the value of multiplex testing and demonstrate that urogenital specimens are suitable for herpesvirus DNA studies. Further research is needed to understand herpesvirus–STI coinfections and their potential impact on urogenital health.

## Conclusions

Our results demonstrate the feasibility of multiplex testing of herpesviruses in urogenital samples. The prevalence of EBV DNA increased among young women with sexually transmissible pathogen coinfection suggesting more frequent shedding of EBV DNA among those with genital infections and likely higher sexual risk-taking behavior.

## Data Availability

The datasets used and/or analysed during the current study are not openly available. The data of this study are available from the Finnish Social and Health Data Permit Authority for researchers who meet criteria for access to confidential data. For instructions, see https://findata.fi/en/. Requests must be addressed through https://asiointi.findata.fi/.

## Abbreviations

CMV: Cytomegalovirus
CNS: Central nervous system
*C. trachomatis*: *Chlamydia trachomatis*
EBV: Epstein–Barr virus
HHV: Human herpesvirus
HPV: Human papillomavirus
HSV: Herpes simplex virus
HSV-1: Herpes simplex virus type 1
HSV-2: Herpes simplex virus type 2
KSHV: Kaposi’s sarcoma–associated herpesvirus
NAAT: Nucleic acid amplification test
*N. gonorrhoea**e*: *Neisseria gonorrhoea* *e*
STI: Sexually transmitted infection
